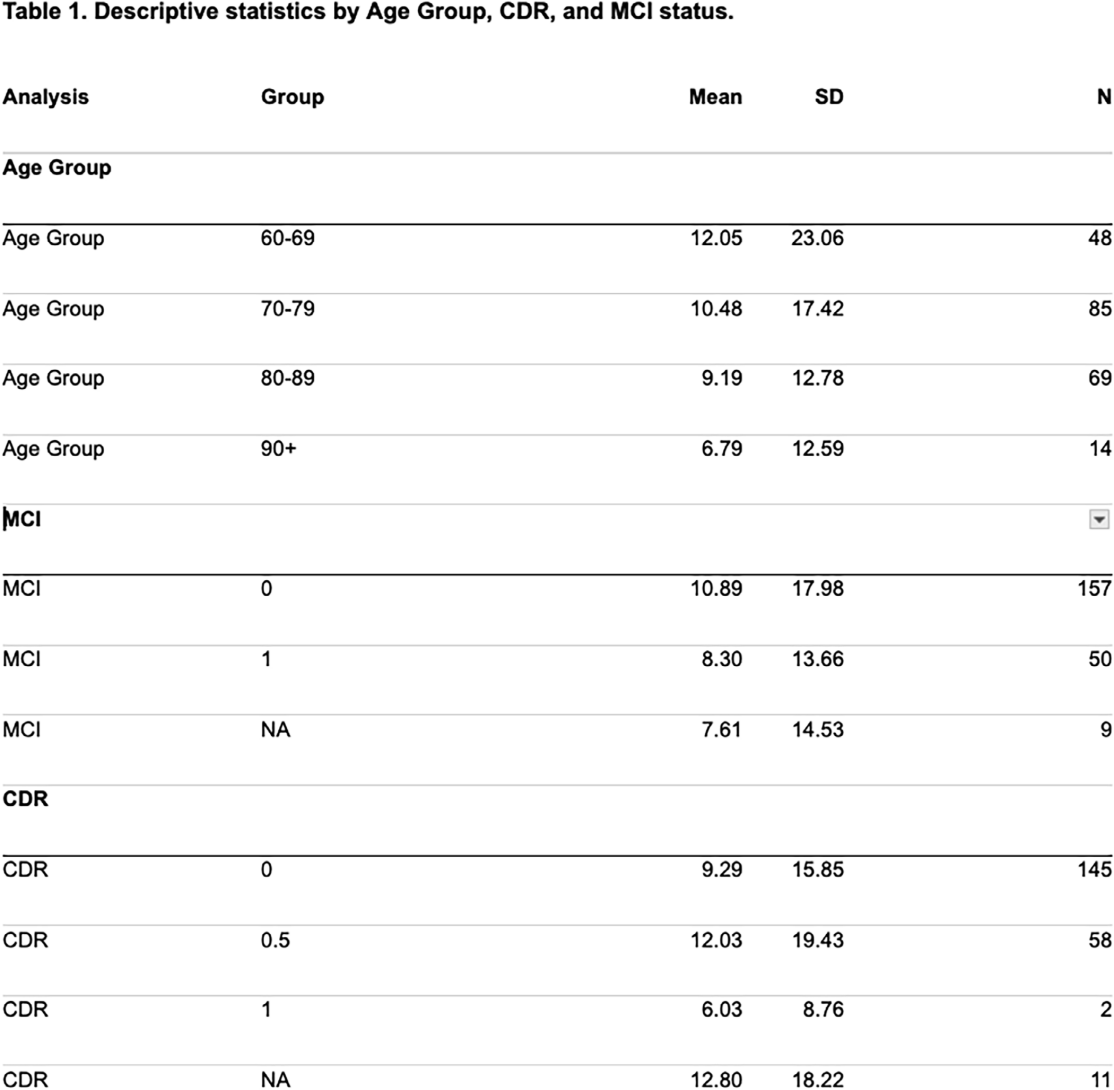# Exposures to PM2.5 in Older Adults: Differences by Cognitive Status and Age in the Einstein Aging Study

**DOI:** 10.1002/alz70856_106788

**Published:** 2026-01-08

**Authors:** Nelson A. Roque, Mindy J. Katz, Angel Garcia De La Garza, Dean Hosgood, Charles B Hall

**Affiliations:** ^1^ The Pennsylvania State University, University Park, PA, USA; ^2^ Albert Einstein College of Medicine, Bronx, NY, USA

## Abstract

**Background:**

Air pollution is a key risk factor for dementia and negatively affects respiratory, cardiovascular, and cognitive health. Older adults are particularly vulnerable due to age‐related changes and cumulative exposure over their lifespan. Limited mobility in older age may lead to more consistent but still harmful exposure. However, personal exposure data in this population, especially at high temporal and spatial resolution, remain scarce.

**Methods:**

We analyzed PM2.5 data from the Atmotube Pro air quality sensor worn by older adults enrolled in the Einstein Aging Study (P01AG003949; N = 209, Mean Age = 77.4, SD = 7.85; 68% female, 14% Hispanic or Latino, 24% MCI). Sensors measure particulate matter (PM1, PM2.5, PM10), VOCs, and meteorological factors every 5 minutes for 14 days annually over five years. This analysis focuses on Year 1 data available so far (January 2025 release), evaluating PM2.5 levels and variability as a function of age and cognitive status (Clinical Dementia Rating, CDR; Mild Cognitive Impairment, MCI).

**Results:**

The raw time‐series data were aggregated at the participant, hour, and day‐of‐week level using median PM2.5 values. These metrics were then summarized by participant demographics and cognitive characteristics (e.g., age, MCI status) to calculate the mean and standard deviation of hourly medians. Group differences in air pollution exposure (mean and variability) are reported in Table 1 below. Median pollution levels and variability were slightly lower in older groups, those with higher CDR scores, and those with MCI, but not significantly.

**Conclusion:**

This study provides high‐resolution air pollution exposure data in older adults, revealing substantial variability not captured by regional monitoring. These findings hint at age‐graded and cognitive‐status graded effects on exposure, and highlight the need to further investigate cognitive effects through neuropsychological testing and smartphone‐based data collection, and for wearable monitoring to improve exposure assessments and inform policies.